# How does the pattern of root metabolites regulating beneficial microorganisms change with different grazing pressures?

**DOI:** 10.3389/fpls.2023.1180576

**Published:** 2023-07-06

**Authors:** Ting Yuan, Weibo Ren, Zhaoming Wang, Ellen L. Fry, Shiming Tang, Jingjing Yin, Jiatao Zhang, Zhenyu Jia

**Affiliations:** ^1^ Inner Mongolia Key Laboratory of Grassland Ecology, School of Ecology and Environment, Inner Mongolia University, Hohhot, China; ^2^ Key Laboratory of Forage Breeding and Seed Production of Inner Mongolia, Inner Mongolia M-Grass Ecology and Environment (Group) Co., Ltd., Hohhot, China; ^3^ Department of Biology, Edge Hill University, Ormskirk, United Kingdom; ^4^ Key Laboratory of Model Innovation in Forage Production Efficiency, Institute of Grassland Research, Chinese Academy of Agricultural Sciences, Hohhot, China

**Keywords:** grazing pressure, *Leymus chinensis*, root metabolites, beneficial rhizobacteria, saprophytic fungi, mycorrhizal fungi

## Abstract

Grazing disturbance can change the structure of plant rhizosphere microbial communities and thereby alter the feedback to promote plant growth or induce plant defenses. However, little is known about how such changes occur and vary under different grazing pressures or the roles of root metabolites in altering the composition of rhizosphere microbial communities. In this study, the effects of different grazing pressures on the composition of microbial communities were investigated, and the mechanisms by which different grazing pressures changed rhizosphere microbiomes were explored with metabolomics. Grazing changed composition, functions, and co-expression networks of microbial communities. Under light grazing (LG), some saprophytic fungi, such as *Lentinus* sp., *Ramichloridium* sp., *Ascobolus* sp. and *Hyphoderma* sp., were significantly enriched, whereas under heavy grazing (HG), potentially beneficial rhizobacteria, such as *Stenotrophomonas* sp., *Microbacterium* sp., and *Lysobacter* sp., were significantly enriched. The beneficial mycorrhizal fungus *Schizothecium* sp. was significantly enriched in both LG and HG. Moreover, all enriched beneficial microorganisms were positively correlated with root metabolites, including amino acids (AAs), short-chain organic acids (SCOAs), and alkaloids. This suggests that these significantly enriched rhizosphere microbial changes may be caused by these differential root metabolites. Under LG, it is inferred that root metabolites, especially AAs such as L-Histidine, may regulate specific saprophytic fungi to participate in material transformations and the energy cycle and promote plant growth. Furthermore, to help alleviate the stress of HG and improve plant defenses, it is inferred that the root system actively regulates the synthesis of these root metabolites such as AAs, SCOAs, and alkaloids under grazing interference, and then secretes them to promote the growth of some specific plant growth-promoting rhizobacteria and fungi. To summarize, grasses can regulate beneficial microorganisms by changing root metabolites composition, and the response strategies vary under different grazing pressure in typical grassland ecosystems.

## 1 Introduction

Grasslands are one of the largest and most important terrestrial ecosystems that not only effectively maintain global ecosystem services but also provide a resource with great potential for food production (Fan et al., 2021). Many grasslands have been degraded due to climate change and improper anthropogenic interference, especially overgrazing (Slimani et al., 2010). Grazing is a common form of land-use worldwide and it drives changes in composition of soil microbial communities (Eldridge et al., 2017). Grazing by herbivores can directly and indirectly affect plant roots and soil microbial community composition by trampling, effects on litter decomposition, and deposition of animal dung and urine (Eldridge and Delgado-Baquerizo, 2017). Moreover, effects vary with different grazing pressures. Light and moderate grazing can improve grassland ecosystem functions by increasing nutrient cycling rates, dry matter production, and energy storage and by facilitating activities of soil microbial communities (Zhao et al., 2017; Chen et al., 2021). By contrast, heavy grazing decreases defoliation and decreases aboveground biomass, resulting in decreased photosynthesis and reduced inputs of belowground carbon (Aldezabal et al., 2015).

Plants and associated microbiomes form a “holobiont”, within which plant–soil microbiome interactions have key roles in nutrient acquisition and stress tolerance (Miyauchi et al., 2020). Environmental stress can lead to changes in plant physiological states and metabolic pathways, which influence the composition of rhizodeposits, including root exudates and complex root tissue compounds. Plants likely adopt the “cry for help” strategy to actively seek cooperation with different microbes to combat environmental stresses and increase resistance, which includes the secretion of root compounds into the rhizosphere (Toju et al., 2018; Li et al., 2022). Plant root exudates comprise primary metabolites such as sugars, amino acids, and organic acids, as well as secondary metabolites such as lipids, flavonoids, terpenes, phytohormones, and alkaloids, all of which are used as an energy source or as a metabolic signal for the recruitment of microorganisms in order to modulate plant performance (Schmidt et al., 2019).

Rhizodeposits attract specific soil microorganisms to colonize roots and rhizospheres and act as signals to mediate positive interactions with beneficial microorganisms such as rhizobia, mycorrhizal fungi, and plant growth-promoting rhizobacteria (PGPR) (Vives-Peris et al., 2020). For example, malic acid can attract specific beneficial bacteria to the rhizosphere (Rudrappa et al., 2008), and specific flavonoid compounds released by some legumes can recruit nitrogen-fixing bacteria (Hassan and Mathesius, 2012). Compounds secreted by roots such as strigolactones are key in promoting colonization of mycorrhizal fungi (Steinkellner et al., 2007). In addition, secretion of antimicrobial compounds, such as coumarin, alters assembly of root-associated microbial communities, triggers induced systemic resistance (ISR), and increases resistance to herbivores (Stringlis et al., 2018). Thus, plants can alter the secretion pattern and composition of root exudates when attacked to regulate beneficial microbes and shape the microbial community.

Corresponding intra-plant metabolic signals between aboveground and belowground parts are used to increase plant resistance (Rasmann et al., 2005). Recent studies have shown that grazing disturbance can significantly alter the structure of plant rhizosphere microbial communities, and feedback acts on the plants to promote its growth (Li et al., 2022). In addition, a negative correlation was found between grazing intensity and root exudates production, and root exudate regulated soil microbial community composition (Zhao et al., 2017). In our previous study, the grass *Leymus chinensis* released metabolic signals to recruit key beneficial bacteria and alleviate grazing stress (Yin et al., 2022).

Leymus chinensis is one of the dominant C3 grasses of the Eurasian steppe, and it is important in maintaining grassland ecosystem functions as well as animal production because of its good palatability (Wang and Ba, 2008). Here, we studied the changes of rhizosphere bacterial and fungal communities of *L. chinensis* under different grazing pressures in typical grasslands, and their relationship with root metabolites. We ask three key research questions. Can plants regulate specific beneficial microorganisms in response to grazing? Is there any difference in the beneficial microbiome regulated by plants between light and heavy grazing? Which metabolites produced by roots are responsible for this change? We hypothesized that plants can regulate beneficial microorganisms by changing root metabolites composition, and the response strategies vary under different grazing pressure in typical grassland ecosystems. The aim of this study was to (i) explore the effects of light and heavy grazing on *L. chinensis* root metabolites, rhizosphere microbial community composition, and soil properties, and (ii) determine the specific functions of *L. chinensis* root metabolites in the regulation of beneficial bacteria and fungi under light and heavy grazing. The changes in *L. chinensis* root metabolites were evaluated using LC-MS and changes in the rhizosphere microbiome using Illumina sequencing.

## 2 Materials and methods

### 2.1 Study site and sampling

The study was conducted in a typical grassland at the Grassland Ecosystem Research Station of Inner Mongolia University (44°15′N, 116°31′E), which was 54 km east of Xilinhot in Inner Mongolia, China. The study area has a semiarid climate, with mean annual temperature of 0.5°C–1.0°C, ranging from −19.03°C in January to 21.38°C in July (Zhang et al., 2020). Mean annual precipitation of 271 mm and 87% of which falls during plant growing season from May to September (Zhang et al., 2020). The site has typical steppe vegetation and is dominated by the grasses *Stipa krylovii* and *L. chinensis* (Yu et al., 2018). The experimental site was freely grazed until 2012 and then fenced for 7 years before conducting this experiment. The experiment included three grazing treatments: no grazing (NG), light grazing (LG), and heavy grazing (HG). Three plots (6 m × 8 m) were randomly established for each treatment, for a total of nine plots. The distance between plots in each treatment was 2 m. The LG and HG plots were grazed by three and six sheep at a time, respectively. This grazing treatment was implemented for 4 hours (from 07:00–11:00 a.m.) at the end of each month from June to August each year and conducted from 2019 to 2021.

Roots and rhizosphere soils of *L. chinensis* were collected from NG, LG, and HG plots in 2021 and used for metabolome analysis and microbial sequencing. Briefly, in each plot, bulk soil of three plants was removed by shaking the plants. The soil that remained tightly adhered to roots (<2 mm in thickness) was defined as rhizosphere soil, which was collected with a hairbrush. The bulk soils associated with rhizosphere soils were also analyzed for soil properties.

### 2.2 Determination of soil properties

After removing coarse roots and gravel, soil samples were analyzed for soil physicochemical properties, soil enzymes, and microbial carbon (MBC) and nitrogen (MBN). Details on methods used to analyze soil enzymes and MBC and MBN can be found in Acosta-Martínez et al. (2010) and Vance et al. (1987). Soil properties, including ammonium N ( ${\text{NH}}_{4}^{\;+}$ -N), nitrate N ( ${\text{NH}}_{4}^{\;+}$ -N), available phosphorus (AP), available potassium (AK), dissolved organic C (DOC), total organic carbon (TOC), total P (TP), total C (TC), total N (TN), Alkaline hydrolysis nitrogen (AN) and pH, were analyzed according to Zhang et al. (2020) and Qi et al. (2020).

### 2.3 DNA extraction, amplification, and sequencing

Genomic DNA was extracted from rhizosphere soil samples with a HiPure Stool DNA Kit (Magen, Guangzhou, China) following the manufacturer’s protocols. The bacterial primers 341F (5′-CCTACGGGNGGC WGCAG-3′) and 806R (5′-GGACTACHVGGGTATCTAAT-3′) were used to amplify the V3–V4 regions (Imparato et al., 2016). The fungal ITS2 region was amplified with the primers ITS3 KYO2 (5′-GATGAAGAACGY AGYRAA-3′) and ITS4 (5′-TCCTCC GCTTATTGATATGC-3′) (Toju et al., 2012). Cycling conditions consisted of 5 min at 95°C; 30 cycles of 1 min at 95°C, 1 min at 60°C, and 1 min at 72°C; and 7 min at 72°C. The purified PCR products from different samples were sequenced by Genedenovo Biotechnology Co., Ltd. (Guangzhou, China). Raw tags were filtered and obtained using FASTP (v 0.18.0). All effective tags of all rhizosphere soil samples were clustered by UPARSE (v 9.2.64), and selected sequences were clustered into operational taxonomic units (OTUs) at 97% similarity.

### 2.4 Metabolite profiling analysis

Root metabolites of *L. chinensis* from NG, LG, and HG treatments were extracted and analyzed as previously described (Fasano et al., 2016). Freeze-dried root samples of *L. chinensis* were put into centrifuge tubes, followed by shaking at 30 Hz for 1.5 min using a Mixer Mill (RetschGmbH, Düsseldorf, Germany) and then overnight extraction at 4°C with 70% aqueous methanol. Samples were centrifuged at 10,000 ×*g* for 10 min and then filtered (SCAA-104, 0.22-μm pore size; Shanghai, China). Last, filtered samples were analyzed with liquid chromatography–tandem mass spectrometry (LC-MS/MS) (HPLC, Shimpack UFLC SHIMADZU CBM30A; Shimadzu Corporation, Tokyo, Japan). Samples were injected into a Waters ACQUITY UPLC HSS T3 C18 column (2.1 mm × 100 mm, 1.8 µm) with a flow rate of 400 µL/min. Metabolites were identified using the public databases LipidMaps (http://www.lipidmaps.org/) and Human Metabolome database (http://www.hmdb.ca/) (Wishart et al., 2012). Differential metabolites were those with variable importance in the projection (VIP) > 1 and *p* < 0.05.

### 2.5 Correlation analysis

To determine whether key differential metabolites influenced microbial communities, correlation analyses were conducted between dominant microbial genera and differential metabolites. Those bacteria and fungi that were significantly enriched after grazing (Least discriminant analysis, LDA scores > 2) were selected as the core microorganisms to correlate with differential metabolites. Pearson correlations were used to analyze relations between root metabolites, soil properties, and rhizosphere bacteria and fungi abundance, with correlations considered significant when the correlation coefficient (*r*) > 0.9 and *p* < 0.05.

### 2.6 Statistical analysis

Statistically significant differences (*p* < 0.05) in soil properties among treatments were evaluated by Student’s *t*-test using SPSS 26. Correlations in co-occurrence networks were obtained using the *“psych”* package in R (version 3.4.3). Correlations between rhizosphere microorganisms and soil properties and root metabolites were tested and plotted using the *“Hmsic”* package in R (version 4.0.2). Root metabolite composition was obtained using PERMANOVA with 999 permutations in R (version 1.8.4). Welch’s *t*-test and Kruskal–Wallis test were used to identify significant differences (*p* < 0.05) in microbial community composition among treatments. PICRUSt software was used to predict KEGG functional profiles of bacterial communities (Langille et al., 2013). The FUNGUIld database has been widely used to predict specific functions of detected fungi (Martínez-Diz et al., 2019).

## 3 Results

### 3.1 Effects of grazing on soil properties

Compared with NG, in LG and HG, soil TOC and ${\text{NH}}_{4}^{\;+}$ -N contents decreased significantly and DOC contents increased significantly (*p* < 0.05). Although there were no significant differences between HG and NG, in LG, Soil Urease (URE) activity decreased significantly and Alkaline hydrolysis nitrogen (AN) contents increased significantly (**Table 1**).

**Table 1:** Effects of different grazing pressure on soil properties.

Variables	Grazing pressure		
	NG	LG	HG
TOC(g·kg^−1^)	28.81 ± 1.84a	20.54 ± 2.31b	19.09 ± 1.20b
AK(mg·kg^−1^)	181.60 ± 14.64	172.54 ± 12.89	181.92 ± 16.29
AN(mg·kg^−1^)	171.30 ± 6.28a	212.31 ± 12.83b	185.27 ± 12.44ab
DOC(g·kg^−1^)	0.03 ± 0.01a	0.04 ± 0b	0.04 ± 0
EC(us·cm^−1^)	29.40 ± 1.67	35.77 ± 4.78	35.00 ± 6.09
URE(mg·kg^−1^·h^−1^)	74.3 ± 6.50a	50.20 ± 2.42b	73.90 ± 7.20a
ACP(mg·kg^−1^·h^−1^)	300.86 ± 17.35	259.24 ± 7.86	269.41 ± 28.33
ALP(mg·kg^−1^·h^−1^)	236.87 ± 46.14	186.20 ± 14.01	210.05 ± 18.39
CAT(g·kg^−1^·min^−1^)	83.18 ± 8.75	93.85 ± 4.77	94.50 ± 5.94
SSC(mg·g^−1^·h^−1^)	2.45 ± 0.06	2.58 ± 0.23	2.06 ± 0.11
MBN(mg·kg^−1^)	52.64 ± 12.90	191.86 ± 140.22	42.32 ± 20.61
MBC(mg·kg^−1^)	645.47 ± 93.93	761.96 ± 233.76	617.66 ± 78.53
SWC (%)	0.91 ± 0.01	0.90 ± 0.01	0.91 ± 0.01
TC(mg·kg^−1^)	17.97 ± 0.75	18.63 ± 0.43	18.40 ± 1.44
TN(mg·kg^−1^)	2.00 ± 0.06	2.13 ± 0.03	2.167 ± 0.18
TP(mg·kg^−1^)	6.84 ± 0.26	7.06 ± 0.14	7.41 ± 0.45
${\text{NH}}_{4}^{\;+}$ -N (mg·kg^−1^)	3.06 ± 0.12a	0.99 ± 0.45b	0.71 ± 0.19b
NO_3_-N(mg·kg^−1^)	5.40 ± 0.22	5.64 ± 0.50	6.45 ± 0.97
AP(mg·kg^−1^)	7.47 ± 0.91	6.60 ± 0.64	9.30 ± 2.53
pH	7.03 ± 0.03	6.91 ± 0.11	7.05 ± 0.05
EOC(mg·kg^−1^)	3.74 ± 0.71	4.90 ± 0.46	4.60 ± 0.40
TC/TN	8.98 ± 0.12	8.74 ± 0.21	8.50 ± 0.03

Values are mean ± standard error (n = 3). Means followed by a same letter are not significantly different at p < 0.05. NG, no grazing; LG, light grazing; HG, heavy grazing; TC, Soil total carbon; TN, Soil total nitrogen; TP, Soil total phosphorus; DOC, Dissolved organic carbon; TOC, Total organic carbon; AN, Alkaline hydrolysis nitrogen; AP, Available phosphorus; AK, Available potassium; SWC, Soil water content; EC, Electrical conductivity; ACP, acid phosphatase; ALP, alkaline phosphatase; ammonium N, ${\text{NH}}_{4}^{\;+}$ -N; nitrate N, ${\text{NO}}_{3}^{\;-}$ N; MBC, Microbial carbon; MBN, Microbial nitrogen; URE, Soil urease; CAT, Catalase; EOC, Easily oxidized carbon; SSC, Soil sucrase.

### 3.2 Effects of grazing on rhizosphere microbial communities

Bacterial and fungal communities were characterized with Illumina HiSeq sequencing. There were 988,425 and 1,093,929 effective tags following 16S rRNA and ITS gene sequencing, respectively, after quality control. Sequences were clustered into 33,600 bacterial OTUs and 8011 fungal OTUs on the basis of 97% similarity.

**Figure 1:**
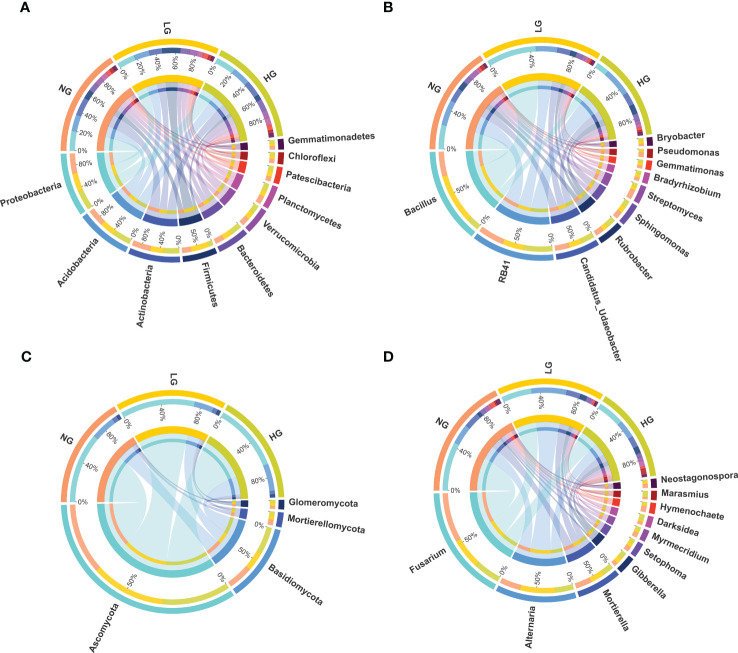
Relative abundances of dominant bacterial phyla **(A)**, bacterial genera **(B)**, fungal phyla **(C)** and fungal genera **(D)** of plant rhizosphere with different grazing treatment. NG, no grazing; LG, light grazing; HG, heavy grazing. The top half and the bottom half circle represents the different samples and the proportions of each dominant phyla in different samples separately.

**Figure 2:**
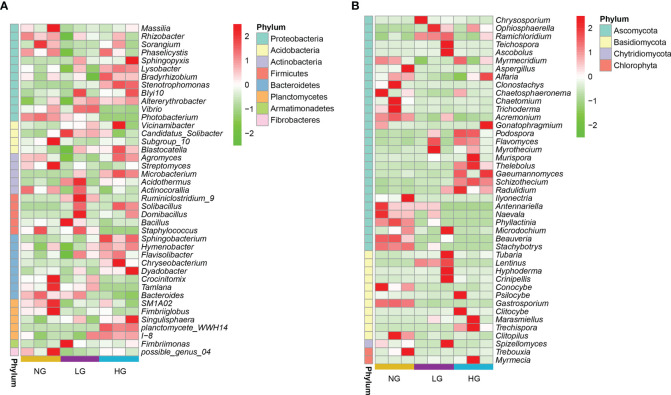
Heat map of the changes in relative abundances of dominant genera of bacteria **(A)** and fungi **(B)** between grazing and no grazing treatments. (LDA score > 2). Colors in the heat map represent the abundance of the genera as indicated by the legend, the redder the color, the higher the abundance and greener the color, the lower the abundance.

Rhizosphere bacterial sequences were assigned to 28 phyla, 253 families, and 394 genera. Bacterial taxonomic composition in different treatments was dominated by members of the phyla Proteobacteria (18.40%), Actinobacteria (16.04%), Acidobacteria (15.09%), Firmicutes (10.00%), Bacteroidetes (9.31%), Verrucomicrobia (7.52%), and Planctomycetes (6.70%) (**Figure 1A**). The most abundant bacterial genera were *Bacillus* (9.39%), *RB41* (8.17%), *Candidatus_Udaeobacter* (4.34%), *Rubrobacter* (2.48%), and *Sphingomonas* (2.19%) (**Figure 1B**). To explore which bacteria were associated to plant roots during grazing, bacteria were identified that were significantly enriched (LDA score > 2). The bacteria *Sphingopyxis* sp., *Lysobacter* sp., *Solibacillus* sp., *Bradyrhizobium* sp., and *Stenotrophomonas* sp. were highly enriched in HG (**Figure S1B**), increasing significantly by 1.15–5.63 times compared with NG (*p* < 0.05, **Figure 2A**). The dominant genera were members of the dominant phyla, such as *Proteobacteria*.

Fungal sequences were assigned to 8 phyla, 154 families, and 244 genera. In all rhizosphere soil samples, the dominant phyla were Ascomycota (68.32%), Basidiomycota (22.71%), Mortierellomycota (4.23%), Glomeromycota (2.67%), Mucoromycota (0.20%), and Chytridiomycota (0.14%) (**Figure 1C**). In Ascomycota and Basidiomycota, the most abundant fungal genera were *Fusarium* (32.67%), *Alternaria* (22.76%), *Mortierella* (12.62%), *Gibberella* (4.92%), *Setophoma* (3.99%), *Myrmecridium* (3.20%), *Darksidea* (3.09%), *Hymenochaete* (2.96%), *Marasmius* (2.51%), and *Neostagonospora* (2.49%) (**Figure 1D**). Similar to bacteria, there were significant differences in relative abundances of core fungal genera (LDA scores > 2) between grazing and no grazing (**Figures S1C, D**). Significant shifts in dominant genera (in Ascomycota and Basidiomycota) occurred in LG and HG, thereby altering fungal communities (**Figure 2B**). Among which, the saprophytic beneficial fungi *Ascobolus* sp., *Lentinus* sp., *Hyphoderma* sp. and *Ramichloridium* sp. were significantly enriched in LG compared with NG.

### 3.3 Analysis of co-occurrence networks

**Figure 3:**
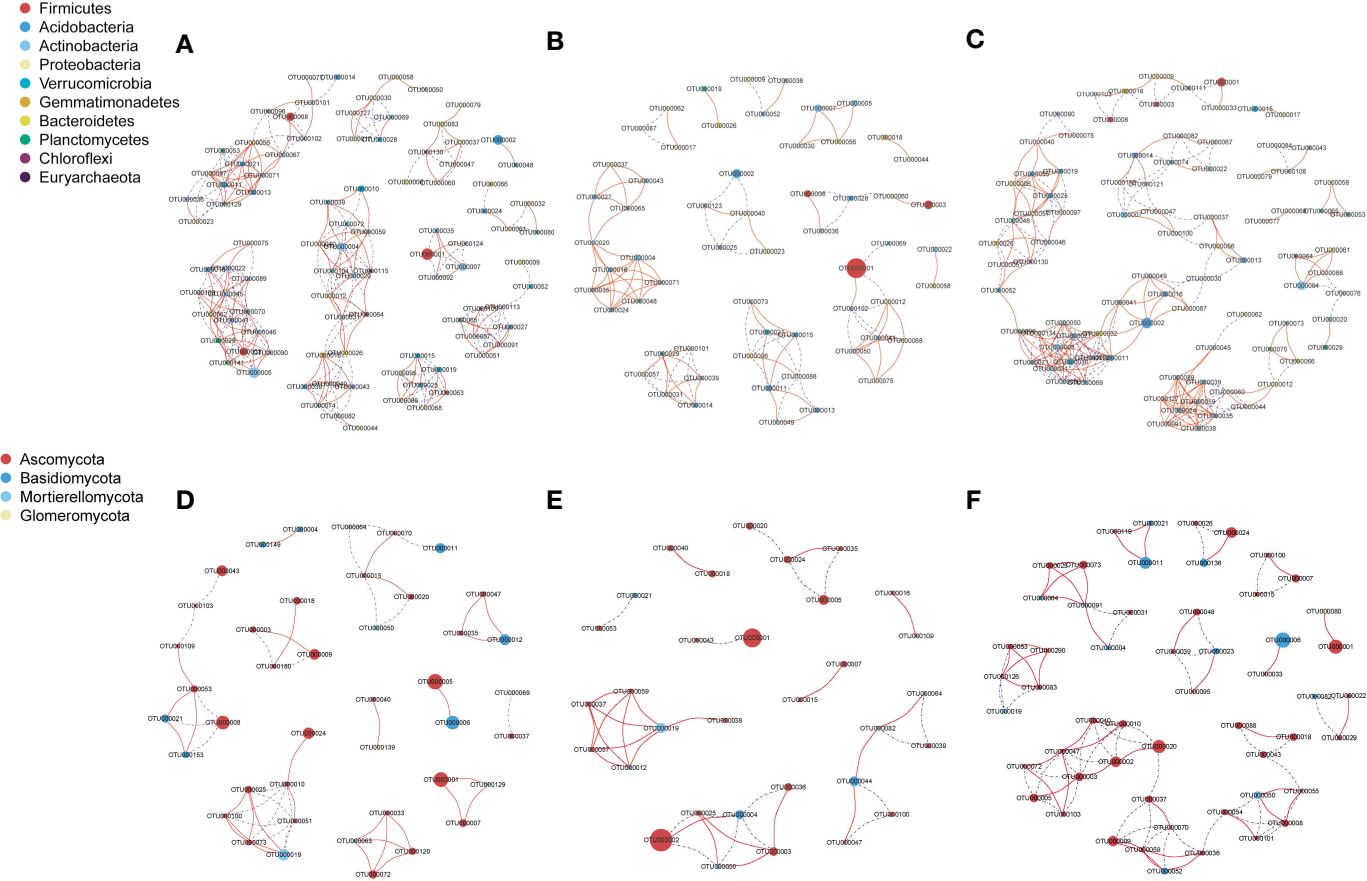
Bacterial (**A**: no grazing, NG; **B**: light grazing, LG; **C**: heavy grazing, HG) and fungal (**D**: NG; **E**: LG; **F**: HG) co-occurrence networks of operational taxonomic units under different grazing treatments. Operational taxonomic units with an relative abundance (RA) > 0.1% in all samples were selected. A connection indicates a strong (Pearson’s r > 0.9 or r <−0.9) and significant (p < 0.05) correlation. Node size represents the relative abundance of microbes, and red and blue lines represent positive and negative correlations, respectively.

The bacterial network in NG included 94 nodes (268 edges), that in LG included 59 nodes (102 edges), and that in HG included 89 nodes (232 edges) (**Figure 3**). However, the fungal network in NG included 42 nodes (52 edges), that in LG included 32 nodes (38 edges), and that in HG included 54 nodes (86 edges). Compared with numbers of correlations in NG (positive: 31; negative: 21), numbers were lower in LG (positive: 23; negative: 15) but higher in HG (positive: 43; negative: 43) (**Table 2**). Moreover, there were fewer connections in bacterial and fungal networks in LG than in NG, indicating that network complexity of bacterial and fungal communities in LG decreased. However, fungal networks were more complex in HG than in NG.

**Table 2:** Properties of the co-occurrence networks of the bacterial and fungal community.

Network properties	Bacteria			Fungi		
Network properties	NG	LG	HG	NG	LG	HG
Number of edges	268	102	238	52	38	86
Number of nodes	94	59	89	42	32	54
Positive edges	134	67	139	31	23	43
Negative edges	134	35	99	21	15	43

Pearson’s r > 0.9 and <−0.9 with a p < 0.05 represented positive and negative correlations, respectively.

### 3.4 Functional Prediction of bacterial and fungal communities

**Figure 4:**
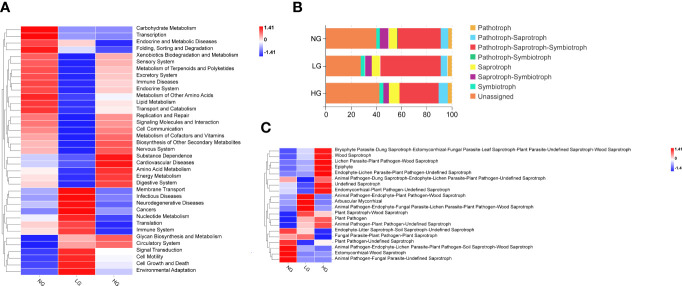
Heatmap showing the RA of prediction function pathways of the rhizosphere bacterial communities **(A)**. The RA of trophic modes **(B)** and the top 20 guilds in fungal communities were assigned by FUNGuild **(C)**. Colors in the heat map **(A, C)** represent the relative abundance of each functional microbial community, with red and blue indicate increased and decreased relative abundance compared with NG, respectively.

According to results of Tax4Fun bacterial functional prediction analysis, pathways associated with Signal transduction, Cell growth and death, Cell motility and Glycan biosynthesis and metabolism were upregulated in both LG and HG compared with NG, and pathways associated with metabolism were identified as the major functional categories. In addition, compared with bacteria in NG, pathways associated with Membrane Transport, Infectious Diseases, Nucleotide Metabolism, and Translation were upregulated in LG and those associated with energy metabolism and amino acid metabolism were upregulated in HG (**Figure 4A**). The prediction results of FUNGuild analysis of nutrient and functional groups of fungal communities identified seven trophic modes and Pathotroph-Saprotroph-Symbiotroph mode accounted for 31.17% to 47.85% of total OTUs. Moreover, compared with NG, the pattern of Pathotroph-Saprotroph-Symbiotroph mode increased in LG but decreased in HG (**Figure 4B**). Among functional guilds, relative abundances of Arbuscular Mycorrhizal Epiphyte in LG were significantly higher than those in NG. Abundances of Plant Saprotroph-Wood Saprotroph and Animal Pathogen-Plant Pathogen-Undefined Saprotroph modes were in higher in both LG and HG than in NG, indicating those modes could have key roles in nutrient cycling and decomposition of organic matter (**Figure 4C**).

### 3.5 Effects of grazing on root metabolites profiles

**Figure 5:**
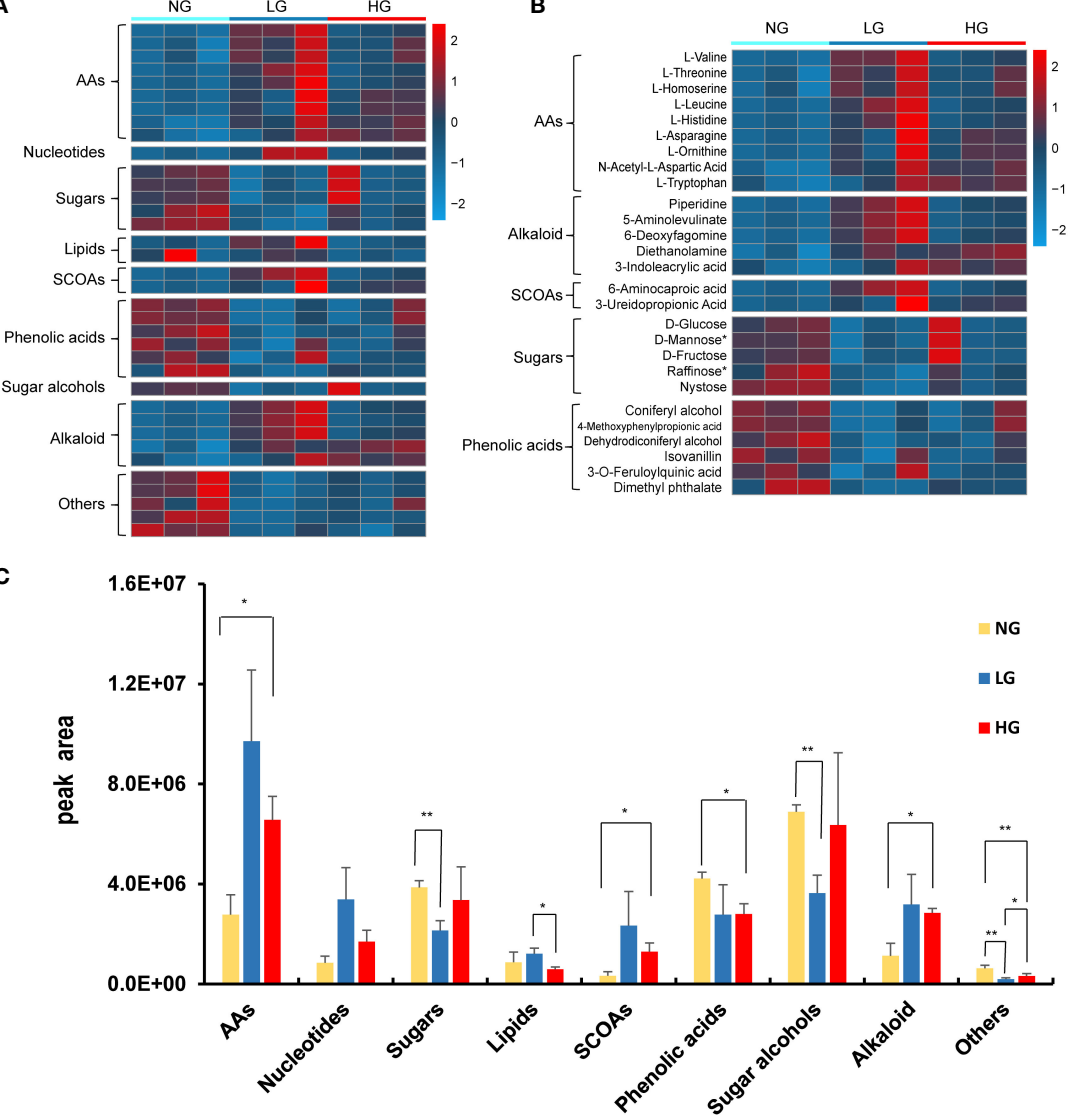
Heatmap of changes in abundance of root metabolites that were significantly different in grazing and no grazing treatments **(A, B)**. Abundance (cumulative peak area) of compound categories of the significantly different root metabolites **(C)**. Each bar represents the average of three replicates. The asterisk indicates statistically significant differences (*t* test, *p* < 0.05) between each root metabolites of control and treatments.

A total of 974 root metabolites were identified, among which 33 metabolites differed significantly between grazing and no grazing treatments (**Figure 5A**). There were 13 down regulated metabolites and 10 upregulated metabolites in LG compared with NG, and there were 7 down-regulated metabolites and 9 up-regulated metabolites under HG treatment compared with NG treatment. Meanwhile, 8 metabolites (Piperidine, 5-Aminolevulinate, L-Threonine, L-Homoserine, L-Leucine, L-Histidine, 6-Methylmercaptopurine and 6-Aminocaproic acid) were significantly enriched only in LG and 7 metabolites (Diethanolamine, 3-Ureidopropionic Acid, L-Asparagine, L-Ornithine, N-Acetyl-L-Aspartic Acid, 3-Indoleacrylic acid and L-Tryptophan) were significantly enriched only in HG compared with NG (**Figures 5A, B**). The results indicated that the composition of metabolites varied with differences in stress treatments. All identified compounds were divided into broad categories on the basis of structure and chemical nature, which included amino acids (AAs, 9 compounds), nucleotides (1 compound), sugars (5 compounds), lipids (2 compounds), short-chain organic acids (SCOAs, 2 compounds), phenolic acids (6 compounds), sugar alcohols (1 compound), alkaloid (5 compounds), and others (5 compounds) (**Figure 5A**). When evaluated at the group level, the abundance of these metabolites varied significantly with grazing treatment, respectively (**Figure 5C**). Overall, AAs, SCOAs and alkaloid were more abundant in root metabolites under LG and HG, whereas phenolic acids and sugars were less abundant (*p* < 0.05) (**Figure 5B**).

### 3.6 Effects of root metabolites and soil properties on rhizosphere bacteria

**Figure 6:**
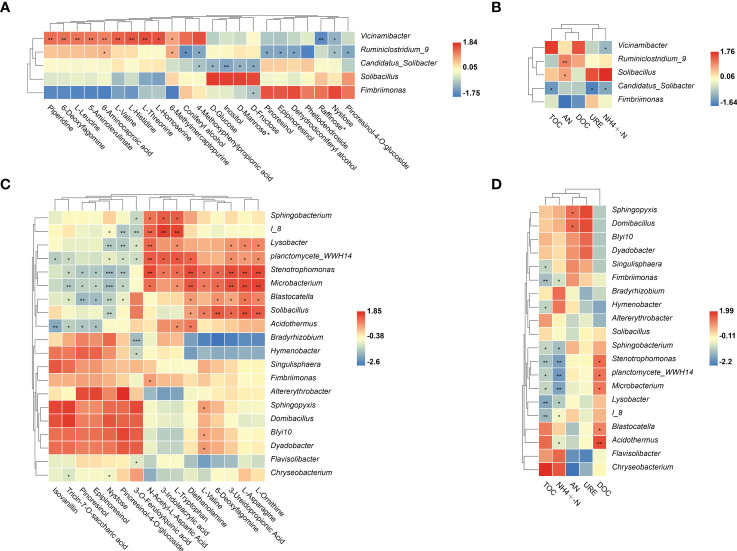
Soil bacteria genus with significant changes in abundance as influenced by differential root metabolites and different soil properties in LG **(A, B)** and HG **(C, D)** treatments. Colors in the heat map represent correlations as indicated by the legend, with red indicating a positive correlation and blue indicating a negative correlation. *p < 0.05, **p < 0.01,***p < 0.001. AN= Alkaline hydrolysis nitrogen; URE= Soil urease; DOC= Dissolved organic carbon; TOC= Total organic carbon; ammonium N= ${\text{NH}}_{4}^{\;+}$ -N.

To determine whether changes in plant root metabolites and soil properties affected rhizosphere microbial colonization, correlation analyses were conducted. In LG, *Vicinamibacter* sp. was significantly positively correlated with almost all significantly enriched metabolites but was not significantly positively correlated with soil nutrients (**Figures 6A, B**). *Ruminiclostridium_9* sp. and *Solibacillus* sp. were significantly positively correlated with AN (**Figure 6B**). Moreover, some significantly enriched bacteria were significantly positively correlated with significantly enriched metabolites (AAs, SCOAs, and alkaloids). In HG, the bacteria *Lysobacter* sp., *Stenotrophomonas* sp., *Microbacterium* sp., and *Planctomycete_WWH14* sp. were significantly positively correlated with AAs (N-Acetyl-L-Aspartic Acid, L-Tryptophan, L-Asparagine and L-Ornithine), and SCOAs (3-Ureidopropionic Acid). Notably, *Stenotrophomonas* sp. were also significantly positively correlated with three alkaloid (3-Indoleacrylic acid, Diethanolamine and 6-Deoxyfagomine) (**Figure 6C**). These metabolites are only significantly enriched in HG treatment compared with NG treatment. Moreover, *Stenotrophomonas* sp.*, Microbacterium* sp. and *Planctomycete_WWH14* sp. were significantly negatively correlated with TOC and ${\text{NH}}_{4}^{\;+}$ -N (**Figure 6D**).

### 3.7 Effects of root metabolites and soil properties on rhizosphere fungus

**Figure 7:**
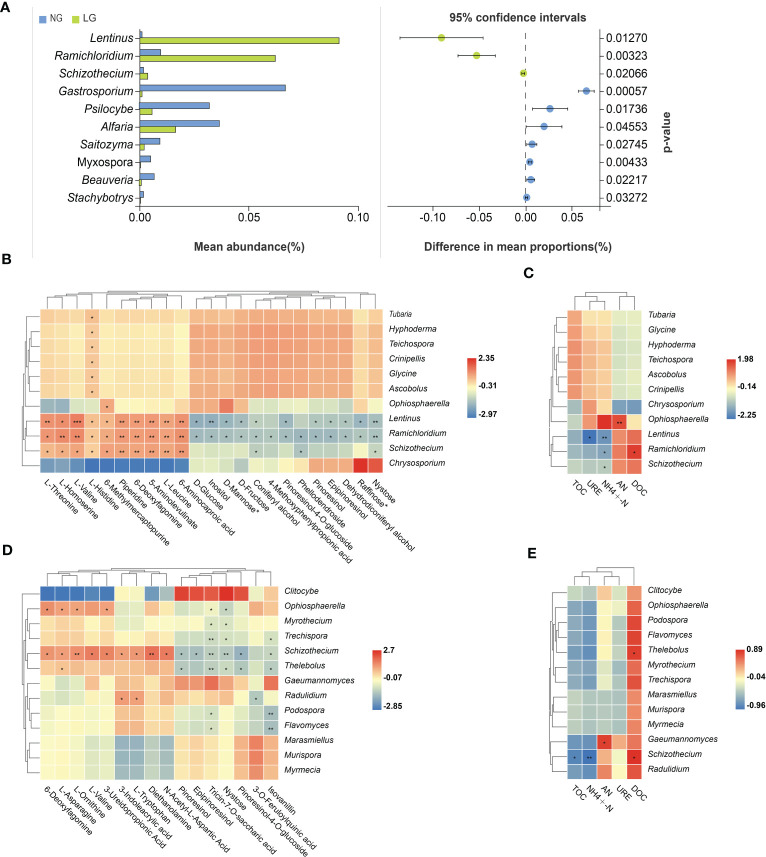
Welch’s t test was used to compare the significant differences in mean abundance between NG and LG **(A)**. Soil fungi genus with significant changes in abundance as influenced by differential root metabolites and different soil properties under LG **(B, C)** and HG **(D, E)** treatment. Colors in the heat map represent correlations as indicated by the legend, with red indicating a positive correlation and blue indicating a negative correlation. *p < 0.05, **p < 0.01. ***p < 0.001. AN= Alkaline hydrolysis nitrogen; URE= Soil urease; DOC= Dissolved organic carbon; TOC= Total organic carbon; ammonium N= ${\text{NH}}_{4}^{\;+}$ -N.

Welch’s *t*-test was used to compare significantly enriched fungi between NG and LG. In LG, the saprophytic fungi *Lentinus* sp., *Ramichloridium* sp., and *Schizothecium* sp. were significantly enriched and were correlated with all significantly enriched metabolites, but were significantly negatively correlated with ${\text{NH}}_{4}^{\;+}$ -N (**Figures 7A–C**). In addition, the saprophytic fungi *Tubaria* sp.*, Hyphoderma* sp.*, Teichospora* sp.*, Crinipellis* sp.and *Ascobolus* sp. were significantly positively correlated with L-Histidinebut were not significantly positively correlated with soil nutrients. In HG, the significantly enriched fungus *Schizothecium* sp. was significantly correlated with DOC and all differential metabolites, except 3-O-feruloylquinic acid, but was significantly negatively correlated with TOC and ${\text{NH}}_{4}^{\;+}$ -N (**Figures 7D, E**).

## 4 Discussion

### 4.1 Effects of grazing on soil microbial communities

The purpose of this study was to determine the interaction between plant roots and soil microorganisms in typical grasslands with different grazing pressures. Our results revealed that grazing changed microbial community composition, but no significant differences were found between grazing and no grazing treatments in alpha diversity of rhizosphere bacteria and fungi. Grazing can affect soil microbial communities in multiple ways, and different levels of grazing pressure have different effects on soil microbial diversity, composition, and structure (Delgado-Baquerizo et al., 2016; Wang et al., 2020). A previous study confirmed that light grazing significantly affected soil fungal community composition but did not affect bacterial community composition in the semiarid grassland ecosystem (Chen et al., 2021). Those results are consistent with the greater numbers of significantly enriched fungal genera than bacterial genera in LG than in NG in this study. Moreover, the numbers of significantly enriched core bacterial genera were greater under HG than under LG, with the dominant bacterial genera were mainly composed with Proteobacteria, Actinobacteria and Acidobacteria, which were significantly enriched(**Figure 2A**). Those key bacterial phyla are associated with plants and contribute to resistance against environmental stresses (Palaniyandi et al., 2013). In addition, light grazing is beneficial to plants that grow slowly and produce low-quality litter, which is conducive to the growth of soil fungi, whereas heavy grazing is beneficial to plants that grow rapidly and produce high-quality litter, which favors soil bacteria (Strickland and Rousk, 2010). Similarly, relative abundances of some dominant fungal genera in the phyla Ascomycota and Basidiomycota shifted significantly in response to LG and HG treatments (**Figure 2B**). The results indicated that Basidiomycota and Ascomycota were dominant phyla of root-related fungi, possibly because of adaptation to grazing stress as well as nutrient limitation, which is consistent with previous studies (Marcos et al., 2019; Chen et al., 2017).

The co-occurrence network analysis of microbial communities indicated that LG significantly reduced the complexity of the bacterial network compared with that in NG (**Figures 3A, B**). The result is consistent with those of previous studies in which bacterial networks were smaller and less connected in grazed soil than in ungrazed soil (Chen et al., 2020). By contrast, the connectivity of fungal networks in HG was more complex than that in NG, whereas changes in the bacterial network in HG were not obvious (**Figure 3**). The results indicated that bacterial communities were more stable than fungal communities in HG. The difference in response between microbes might be because heavy grazing reduces the availability of soil C for microbial colonization. Fungi are more dependent on the soil C matrix than bacteria, and therefore, under C limitation with heavy grazing, competition for rhizosphere soil niches among fungi increases (Chen et al., 2018). Heavy grazing decreases plant biomass and root productivity, which leads to a reduction in soil organic C (Wang et al., 2017). Similarly, in this study, a significant reduction in TOC was observed in LG and HG compared with that in NG (**Table 1**), which is consistent with previous findings that TOC decreases with an increase in grazing pressure (He et al., 2008). As a result of the reduction in TOC, competition within the fungal community for the soil C matrix increases, thereby decreasing community stability under adverse environmental conditions (Newman, 2006).

Notably, the effect of grazing on bacterial networks was much greater than that on fungal networks, which is a result similar to those in a previous study that found bacterial communities have greater network complexity than that of fungal communities (Wang et al., 2021). The cooperative relations among members of a bacterial network are stronger than those of a fungal network in the soil of a grazed grassland, indicating increased stability and tolerance to grazing disturbance (Zhang et al., 2018). In addition, livestock consume nearly half of aboveground biomass and return it as manure rich in available nutrients and organic substrate, which can be conducive to bacterial growth (Bagchi and Ritchie, 2010).

According to Tax4Fun analysis of bacteria, pathways associated with Amino acid metabolism and Energy metabolism were upregulated in the bacterial function prediction analysis of HG treatment compared with NG treatment (**Figure 4A**). The results are similar to those obtained in a study of bacterial functions in wheat, barley, and tomato soils (Pii et al., 2016; Qu et al., 2021), which is that the most representative pathways of rhizosphere soil after stress treatment are all amino acid metabolism and energy metabolism. After some metabolites associated with amino acid metabolism and energy metabolism being exuded, it is most likely used by soil microorganisms as C and N source (Pii et al., 2015). These data further confirm that the rhizosphere microbial community respond to the plants by modifying their metabolic activities, aiming at promoting bacterial growth by, suggesting that root metabolites affect bacterial community composition and function as C and N sources. Thus, as one of the main signaling molecules and supplies of nutrients in HG, AAs might be crucial in reshaping bacterial communities under heavy grazing.

The fungal Pathotroph-Saprotroph-Symbiotroph mode increased in LG but decreased in HG compared with that in NG (**Figure 4C**). The result indicated that LG promoted synergistic interactions among Pathotroph-Saprotroph-Symbiotroph (Bello et al., 2021). Saprotrophic fungi can promote litter decomposition and nutrition cycling rates (Wang et al., 2022). In this study, compared with NG, grazing resulted in significant increases in relative abundances of saprophytic fungi, which can promote nutrient cycling and formation of soil organic matter (Zhang et al., 2019). Among functional guilds, relative abundances of Arbuscular Mycorrhizal in LG treatment were significantly higher in LG than in NG. Mycorrhizal fungi relieve C restrictions on saprophytic microorganisms, stimulate the growth of saprophytic microorganisms and the production of extracellular enzymes, and accelerate the decomposition of litter and organic matter through mycelial secretions or turnover of biomass. Moreover, C released after arbuscular mycorrhizal fungi infect roots induces changes in rhizosphere bacterial and fungal communities and stimulates extracellular enzyme production of saprophytic microorganisms to obtain N and P (Toljander et al., 2007). These processes indicate that arbuscular mycorrhizal fungi might provide additional C and energy to saprophytic microbes.

### 4.2 Plant–rhizosphere microbial interactions

#### 4.2.1 Plant root metabolites regulate rhizosphere beneficial fungi to promote plant growth under light grazing

In grassland ecosystems, many saprophytic fungi in the Ascomycota promote the mineralization of nutrient elements in soil and thus increase nutrient availability and uptake to promote plant growth (Barnes et al., 2018; Bastida et al., 2019). Symbiotic fungi are primarily mycorrhizal fungi, and mycorrhizae can indirectly interact with saprophytic microorganisms *via* stimulating saprophytic activity and nutrient competition to affect litter decomposition as well as improve host plant resistance and adaptability (Frew et al., 2018). In the analysis of fungal community composition in this study, the saprophytic fungi *Tubaria* sp., *Hyphoderma* sp., *Teichospora* sp., *Crinipellis* sp., *Ascobolus* sp., and *Lentinus* sp. and the mycorrhizal fungus *Schizothecium* sp. were significantly enriched under LG (**Figure 2B**).

*Schizothecium* sp., *Ascobolus* sp., *Lentinus* sp., and *Hyphoderma* sp. are involved in plant litter decomposition, including decomposition of herbaceous stems, wood, and dung, and therefore are important ecosystem saprophytes in helping to recycle nutrients in animal dung (Barron, 2003; Lakshmanan et al., 2008; Mumpuni et al., 2020). *Ramichloridium* sp. have potential in the control of diseases and in the promotion of plant growth (Peters et al., 2020). Moreover, certain active fungi, such as the mycorrhizal fungus *Schizothecium* sp., colonize soils and are involved in the assimilation of root exudate, indicating specific fungi–plant root associations (Hugoni et al., 2018). *Schizothecium* sp. also has biocontrol activity against *Fusarium* wilt (Santoyo et al., 2021). Therefore, saprophytic and mycorrhizal fungi are two important fungal groups involved in most litter decomposition and nutrient cycling processes. In this study, saprophytic and mycorrhizal fungi promoted nutrient cycling and litter decomposition in the rhizosphere soil under LG (Lindahl et al., 2007).

Compounds secreted by roots can promote the establishment of mutualistic relations with different fungi. Beneficial fungi are attracted by specialized plant metabolites, such as sugars, organic acids, hormones, AAs, and antimicrobial compounds, which are used by fungi as sources of energy (Devi et al., 2020; Yadav et al., 2020). Such fungi provide antagonist compounds to improve plant growth and increase nutrient availability as well as stress tolerance. For example, arbuscular mycorrhizal fungi and rhizobium bacteria can be recruited by signaling compounds secreted by plant roots (Besserer et al., 2006). Furthermore, root secretions can affect the metabolic activity of some fungi (de Graaff et al., 2010). Plants produce defensive compounds, nutrients, and signaling molecules during AAs metabolism that interact with microorganisms; in particular, AAs may be crucial in establishing symbiotic interactions (Moormann et al., 2022). Abiotic stress can result in increased exudation of proline and L-theanine, which can be used as energy sources to recruit dominant microbes (Vives-Peris et al., 2020; Xie et al., 2022). Similarly, L-Histidine is essential for plant growth and development, as well as inducing resistance to bacterial pathogens (Seo et al., 2016). Changes in rhizosphere microbial community assembly may be caused by root exudates, but our results only analyzed the differential root metabolites and found that some differential metabolites were significantly enriched after LG (**Figure 5A**), and these differential metabolites may be secreted through the root system and affect microbial community assembly. In addition, some compounds detected in root exudates are usually synthesized at the roots and 63–85% of root metabolites were found in rice root exudates (Mclaughlin et al., 2023). In Our study, beneficial saprophytic and mycorrhizal fungi were significantly positively correlated with L-Histidine (**Figure 7B**), which suggested these significantly enriched beneficial saprophytic and mycorrhizal fungi may be caused by these differential root metabolites, especially L-Histidine, and the saprotrophic fungi gained energy with consumption of the root metabolite (Buée et al., 2009). Therefore, it is inferred that the root system actively regulates the synthesis of these root metabolites under grazing interference, and then secretes them to regulate some specific rhizosphere beneficial fungi and thereby promote litter decomposition, fecal decomposition, and the absorption of soil mineral nutrients.

In this study, most soil properties were not significantly correlated with fungi, likely because host roots maintained a relatively stable feeding environment and feedback interactions with fungi. Because of the root microenvironment had undergone strong environmental filtration and the alteration of soil properties had a low effect on the alteration of fungi community in roots (Beck et al., 2015). Therefore, under light grazing, it is inferred that root metabolites, especially AAs such as L-Histidine, may regulate specific saprophytic fungi to participate in material transformations and the energy cycle and ultimately promote plant growth in grassland ecosystems (Guyonnet et al., 2017).

#### 4.2.2 Root metabolites promote the growth of beneficial rhizosphere growth-promoting bacteria and fungi to relieve the stress of heavy grazing

Plant metabolites have crucial effects in shaping root microbiomes (Jacoby et al., 2021). Plants selectively recruit beneficial rhizosphere microbes by releasing specific metabolites, which may be very helpful in the fight against biotic and abiotic stresses (Qu et al., 2020). The potentially beneficial bacteria *Lysobacter* sp., *Stenotrophomonas* sp., *Microbacterium* sp., and *Planctomycete_WWH14* sp. and the beneficial mycorrhizal fungus *Schizothecium* sp. were significantly enriched in HG compared with NG (**Figures 2A, B**). *Lysobacter* sp. and *Stenotrophomonas* sp. are in the family Xanthomonadaceae, which has members that can directly inhibit pathogens with the secretion of secondary metabolites (Brescia et al., 2020; Deng et al., 2022). Berendsen et al. (2018) reported that foliar infection with a biotrophic pathogen systemically signals to *Arabidopsis thaliana* to promote the growth of *Microbacterium* sp., *Xanthomonas* sp., and *Stenotrophomonas* sp. in the rhizosphere, and collectively, the three bacteria can induce systemic resistance against pathogens. Moreover, recruitment of *Stenotrophomonas* sp. can regulate plant defenses (Liu et al., 2021). In addition, the beneficial mycorrhizal fungus *Schizothecium* sp. has potential biocontrol activity (Santoyo et al., 2021). Therefore, the potentially beneficial microbes may prime plant defense signaling pathways and consequently ameliorate plant stresses. According to the findings of this study, under heavy grazing, plants may regulate specific microbes to increase defensive capabilities.

Specialized plant metabolites such as flavonoids, organic acids, AAs, hormones, and triterpenoids can be used as signal molecules, stimulants, and attractants to shape the composition of microbial communities (Baetz and Martinoia, 2014). For example, small-molecule organic acids can recruit Comamonadaceae (Wen et al., 2020). In addition, *A. thaliana* can recruit and stimulate specific *Pseudomonas* populations by secreting long-chain OAs and AAs to cope with pathogens (Yuan et al., 2018). The *Pseudomonas* can then activate induced systemic resistance and protect plants from pathogens (Wen et al., 2021). A recent study has shown that some compounds detected in root exudates are usually synthesized at the roots and most root metabolites were found in plant root exudates (Mclaughlin et al., 2023). Therefore, in our study, we conclude that part of the metabolites in root metabolites may be secreted outside the roots and thus affect the assembly of soil microbial communities. In addition, root metabolites can selectively regulate the growth of *Arabidopsis* root bacteria from different taxa by acting as antibiotics or proliferating agents (Huang et al., 2019). Similarly, in this study, AAs, SCOAs, and alkaloids were the most abundant root metabolites, whereas sugars and phenolic acids were the least abundant in both LG and HG (**Figure 5B**), which may be because more complex amino acids and alkaloids produce more selective effects (Yuan et al., 2018). In addition, these low molecular weight AAs (N-Acetyl-L-Aspartic Acid, L-Tryptophan, L-Asparagine and L-Ornithine), SCOAs (3-Ureidopropionic Acid) and alkaloid (Diethanolamine and 3-Indoleacrylic acid) were positively correlated with several potentially beneficial rhizobacteria (**Figure 6C**), indicating that these significantly enriched beneficial rhizobacteria changes may be caused by these differential root metabolites and those metabolites may be secreted acting as nutrients and energy sources for specific microorganisms (Bais et al., 2006).

Tryptophan (Trp) has an essential role in regulating plant growth, development, and defense (Ryu and Patten, 2008). In this study, Trp was positively correlated with six potentially beneficial rhizobacteria (**Figure 6C**), indicating that Trp may be secreted by roots and as a signaling molecule to effect beneficial rhizobacteria. Similarly, Diethanolamine, 6-Deoxyfagomine and 3-Indoleacrylic acid were also positively correlated with the enriched bacteria. This result is inconsistent with those of a previous study in which *Ps. camelliae-sinensis* inhibited secretion of alkaloids, organic acids, and AAs in tea seedlings but induced secretion of phenolic acids and flavonoids (Wang et al., 2021). Such differences may be related to metabolic differences between plant species (Wang et al., 2016). Therefore, to help alleviate heavy grazing stress, it is inferred that the root system actively regulates the synthesis of these root metabolites such as AAs, SCOAs, and alkaloids under grazing interference, and then secretes them to promote the growth of some specific plant growth-promoting rhizobacteria and fungi. We will collect root exudates and compare the differences between root exudates and root metabolites, and then conduct further verification tests in the future.

## 5 Conclusion

**Figure 8:**
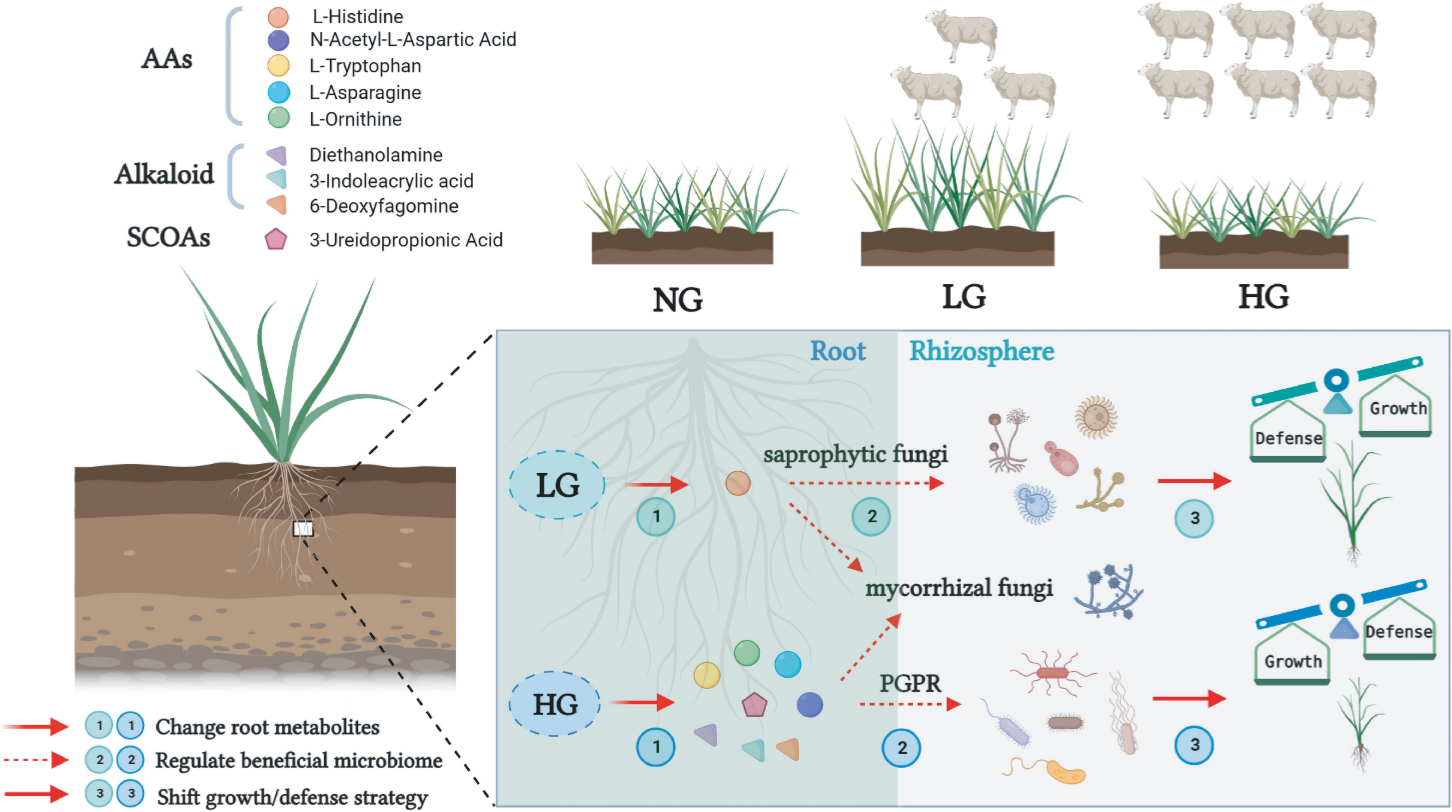
Mechanistic model of beneficial microbial regulation by root metabolites under different grazing pressures. Under light grazing, the root metabolite L-Histidine may regulate specific saprophytic fungi to participate in the energy cycle of a grassland ecosystem and promote plant growth. Under heavy grazing, plants may promote the growth of plant growth-promoting rhizobacteria and beneficial mycorrhizal fungi by amino acids (AAs), short-chain organic acids (SCOAs), and alkaloids to help alleviate the stress and improve plant defenses.

In this study, plants root exude specific metabolites to selectively regulate functional rhizosphere bacteria and fungi from the soil environment. These increased plant growth and protected plants by modulating plant defense capacity to help plants resist grazing stresses and thereby enhancing their ability to adapt to the environment (**Figure 8**). Grassland plants responded to grazing by actively regulating the synthesis of root metabolites under grazing interference, and then secreting them to regulate specific rhizosphere microorganisms, although the response strategies varied under different grazing pressures. Under light grazing, it is inferred that root metabolites, especially AAs such as L-Histidine, may regulate specific saprophytic fungi to participate in material transformations and the energy cycle and ultimately promote plant growth. Furthermore, to help alleviate the stress of heavy grazing and improve plant defenses, it is inferred that the root system actively regulates the synthesis of these root metabolites such as AAs, SCOAs, and alkaloids under grazing interference, and then secretes them to promote the growth of some specific plant growth-promoting rhizobacteria and fungi.

## Data availability statement

The high-throughput sequencing raw data of bacteria and fungi presented in the study are deposited in the NCBI database with SRA accession numbers PRJNA952153 (https://ncbi.nlm.nih.gov/bioproject/PRJNA952153/) and PRJNA952182 (https://ncbi.nlm.nih.gov/bioproject/PRJNA952182/).

## Author contributions

WR: Conceptualization, methodology, software, writing – original draft preparation, writing – review and editing, supervision. TY: Conceptualization, methodology, software, investigation, data curation, writing – original draft preparation, visualization. ST and ZW: Data Curation, software, formal analysis, visualization. JY and ZJ: Supervision, writing – review and editing. EF and JZ: Conceptualization, writing – review and editing, supervision, project administration, funding acquisition. All authors contributed to the article and approved the submitted version.

## Supplementary material

Supplementary Figure 1 Least discriminant analysis (LDA) effect size taxonomic cladogram comparing bacteria **(A, B)** and fungal **(C, D)** under LG and HG treatment. The different classification levels are presented from the inside to the outside. Red nodes indicate enriched genera in LG and HG treatment, yellow nodes indicate no difference, and green nodes indicate enriched genera in NG treatment.
