# Supplementary figures and images for: How does the pattern of root metabolites regulating beneficial microorganisms change with different grazing pressures?
Source: Front Plant Sci. 2023 Jul 6;14:1180576. doi: 10.3389/fpls.2023.1180576 (PMC10361787; doi:10.3389/fpls.2023.1180576)

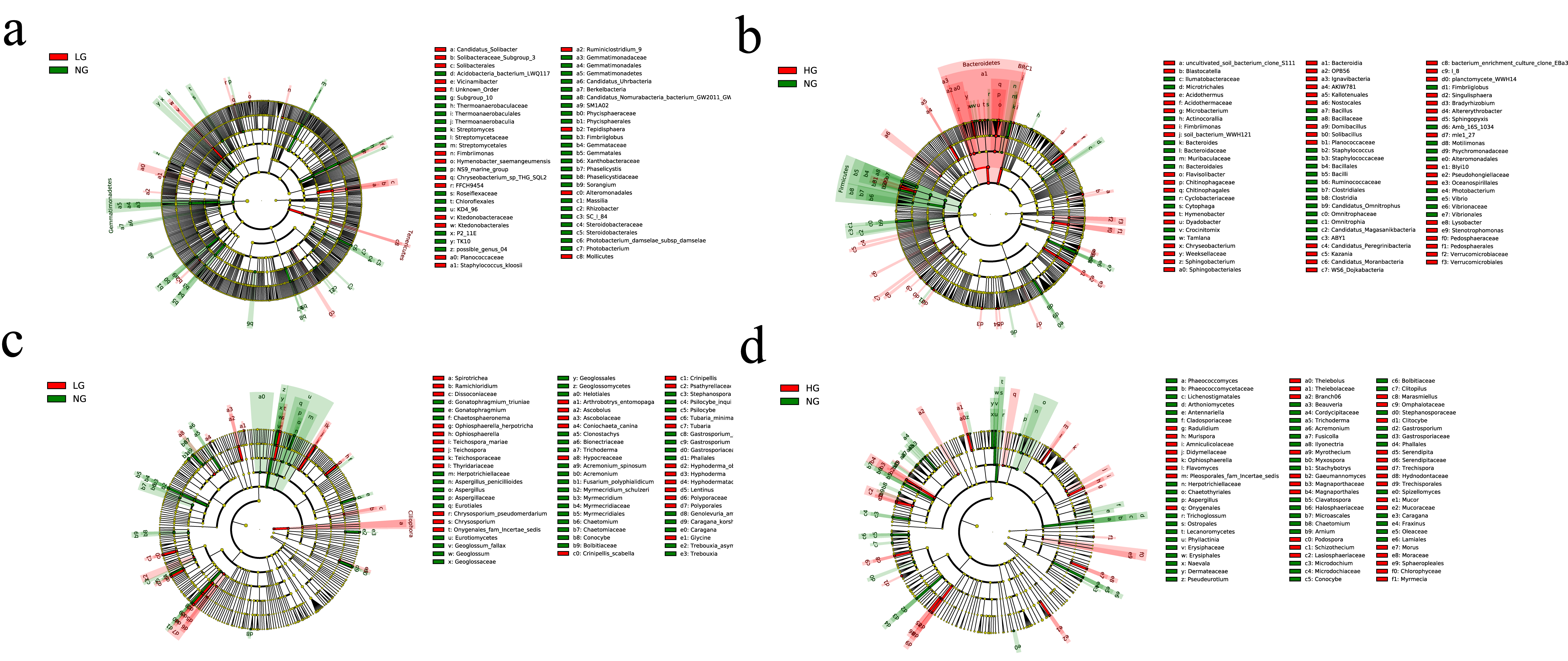

Supplement: Supplementary Figure 1 — Least discriminant analysis (LDA) effect size taxonomic cladogram comparing bacteria (A, B) and fungal (C, D) under LG and HG treatment. The different classification levels are presented from the inside to the outside. Red nodes indicate enriched genera in LG and HG treatment, yellow nodes indicate no difference, and green nodes indicate enriched genera in NG treatment. [file Image_1.jpeg]
