# Supplementary material for: How does the pattern of root metabolites regulating beneficial microorganisms change with different grazing pressures?
Source: Front Plant Sci. 2023 Jul 6;14:1180576. doi: 10.3389/fpls.2023.1180576 (PMC10361787; doi:10.3389/fpls.2023.1180576)
Supplement: Supplementary file 2 [file Table_1.docx]

**Table 1** Effects of different grazing pressures on α diversity of rhizosphere bacteria

| Treatment | sobs | shannon | simpson | chao |
| --- | --- | --- | --- | --- |
| NG | 3765.33±55.90 | 9.49±0.11 | 0.99±0 | 3951.83±55.82 |
| LG | 3578.67±443.69 | 8.90±1.20 | 0.97±0.05 | 3791.40±443.85 |
| HG | 3856.00±241.63 | 9.76±0.14 | 1.00±0 | 4042.80±250.44 |

Notes: Values are mean ± standard error (n = 3).
