# Supplementary material for: How does the pattern of root metabolites regulating beneficial microorganisms change with different grazing pressures?
Source: Front Plant Sci. 2023 Jul 6;14:1180576. doi: 10.3389/fpls.2023.1180576 (PMC10361787; doi:10.3389/fpls.2023.1180576)
Supplement: Supplementary file 3 [file Table_2.docx]

**Table 2** Effects of different grazing pressures on α diversity of rhizosphere fungi

| Treatment | sobs | shannon | simpson | chao |
| --- | --- | --- | --- | --- |
| NG | 868.67±12.01 | 6.36±0.200 | 0.97± | 993.32±33.68 |
| LG | 855.67±13.61 | 5.81±1.31 | 0.92±0.09 | 982.57±24.85 |
| HG | 946±42.23 | 6.59±0.41 | 0.97±0.01 | 1051.02±52.23 |

Notes: Values are mean ± standard error (n = 3).
